# Supplementary figures and images for: Transcriptomic characterization of signaling pathways associated with osteoblastic differentiation of MC-3T3E1 cells
Source: PLoS One. 2019 Jan 4;14(1):e0204197. doi: 10.1371/journal.pone.0204197 (PMC6319725; doi:10.1371/journal.pone.0204197)

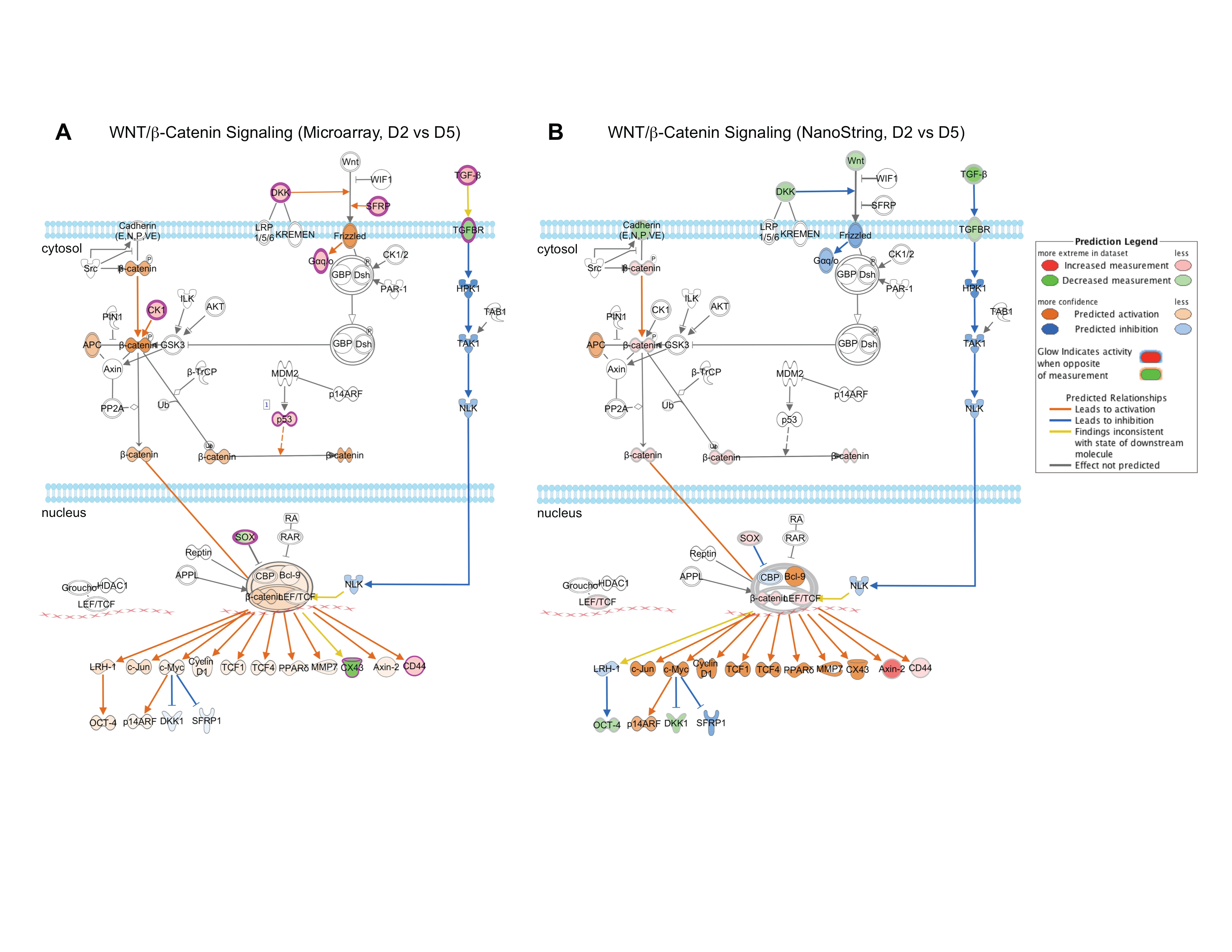

Supplement: S1 Fig — Observed Day 2 to Day 5 changes in expression ratios were used to predict WNT/β-catenin pathway activity using the IPA molecular activity predictor tool. A. Pathway activity prediction based on the microarray dataset. B. Pathway activity based on the NanoString dataset. Observed increases (red) and decreases (green) in mRNA abundance are indicated, as are predicted activation (orange) and inhibition (blue) of downstream targets. (TIFF) [file pone.0204197.s001.tiff]

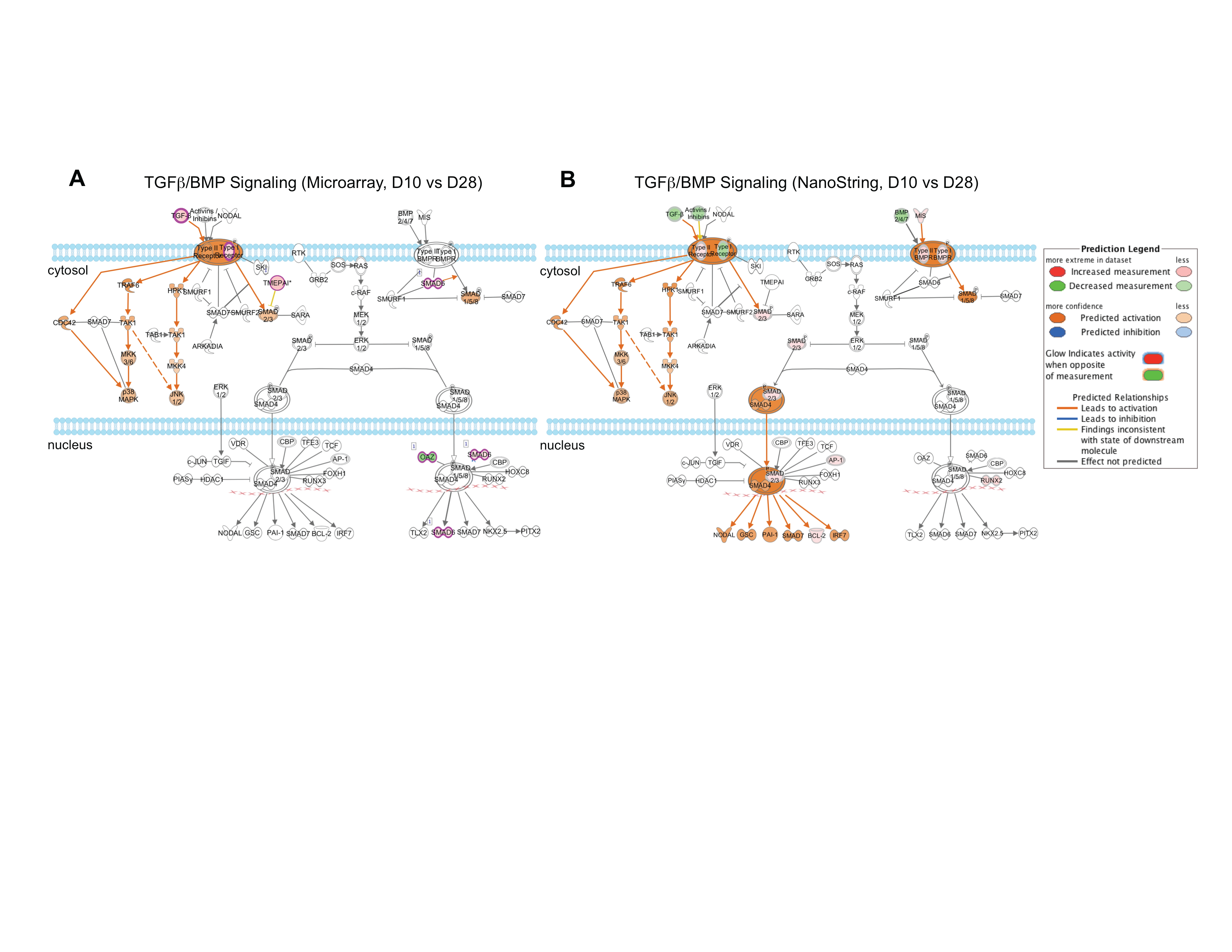

Supplement: S2 Fig — Observed Day 10 to Day 28 changes in expression ratios were used to predict TGFβ/BMP pathway activity using the IPA molecular activity predictor tool. A. Pathway activity prediction based on the microarray dataset. B. Pathway activity based on the NanoString dataset. Observed increases (red) and decreases (green) in mRNA abundance are indicated, as are predicted activation (orange) and inhibition (blue) of downstream targets. (TIFF) [file pone.0204197.s002.tiff]

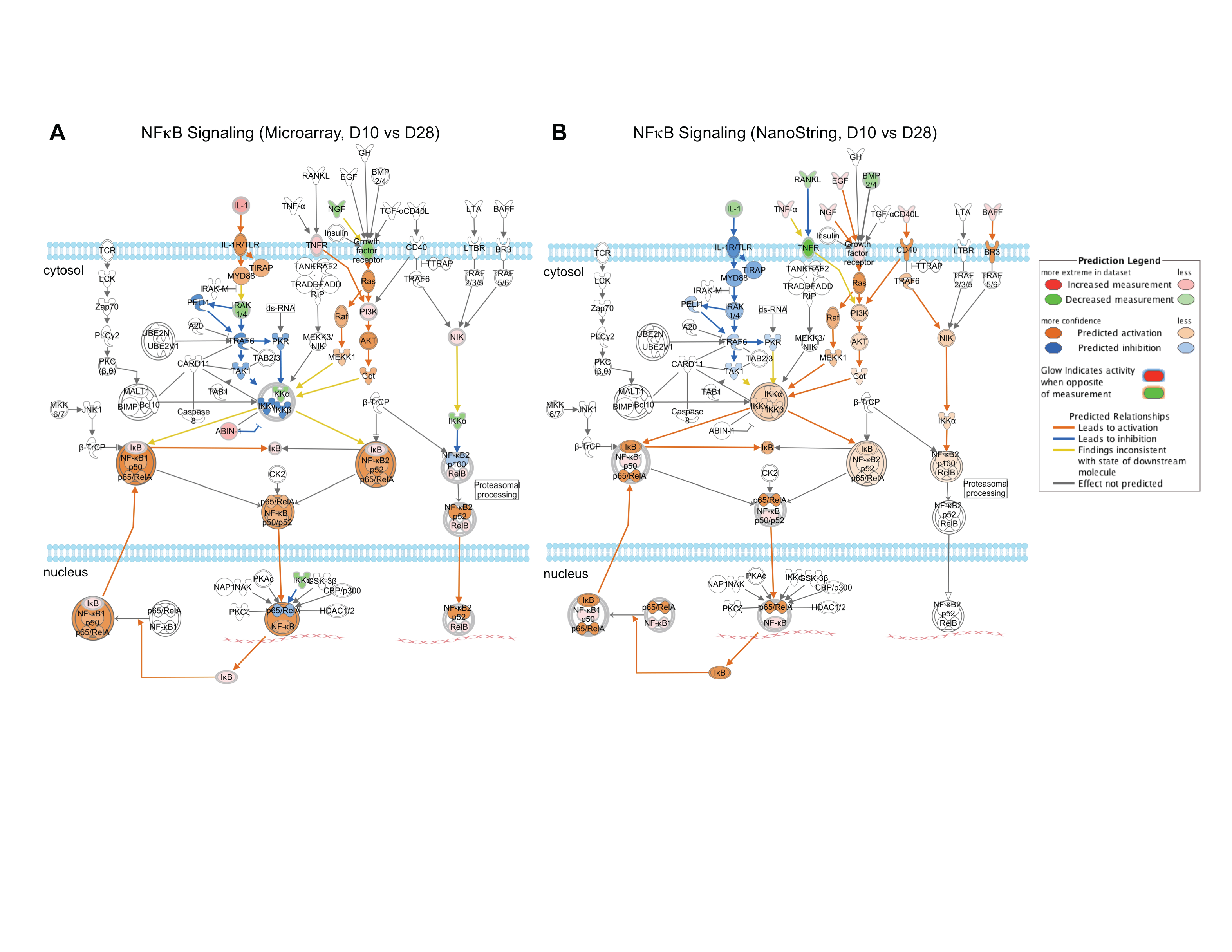

Supplement: S3 Fig — Observed Day 10 to Day 28 changes in expression ratios were used to predict NFκB pathway activity using the IPA molecular activity predictor tool. A. Pathway activity prediction based on the microarray dataset. B. Pathway activity based on the NanoString dataset. Observed increases (red) and decreases (green) in mRNA abundance are indicated, as are predicted activation (orange) and inhibition (blue) of downstream targets. (TIFF) [file pone.0204197.s003.tiff]

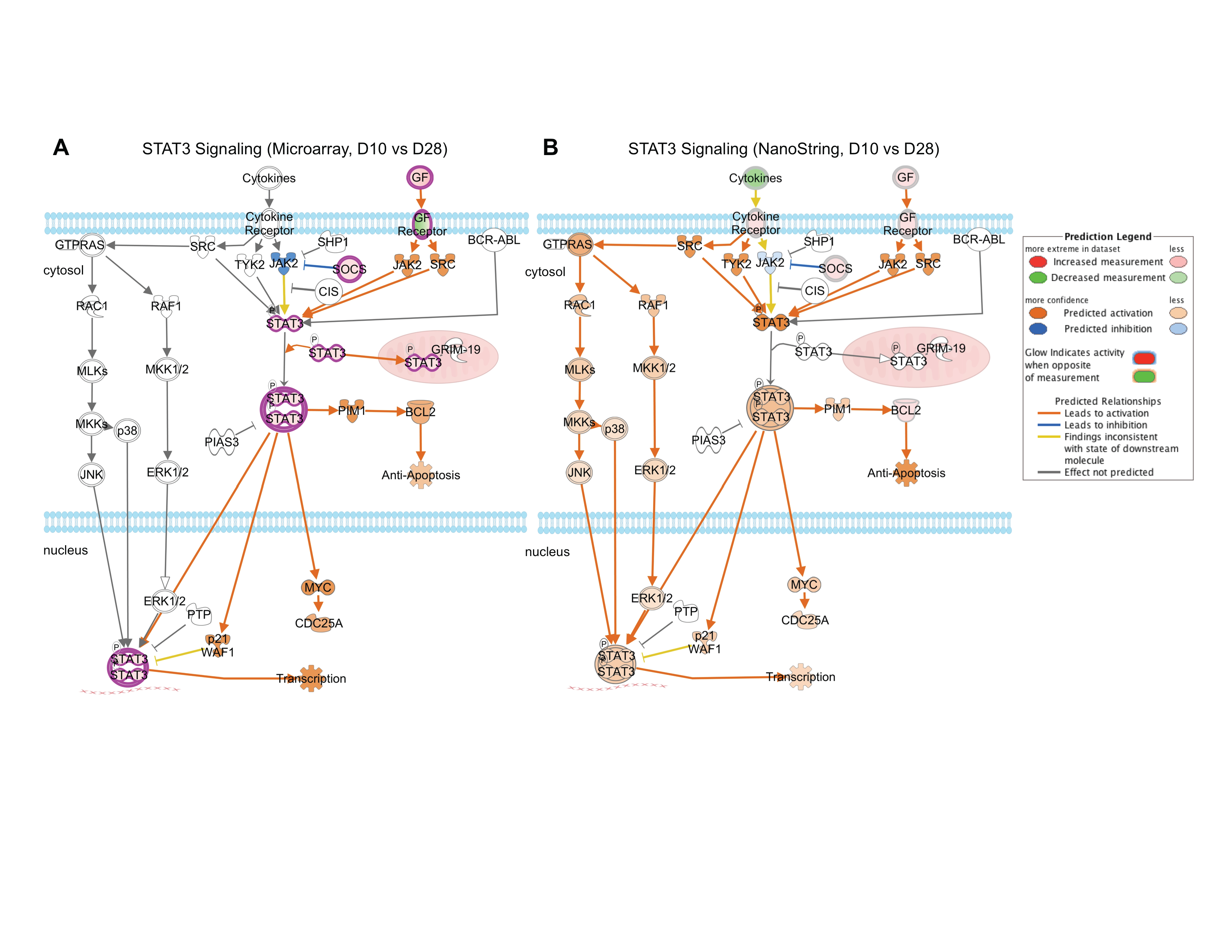

Supplement: S4 Fig — Observed Day 10 to Day 28 changes in expression ratios were used to predict STAT3 pathway activity using the IPA molecular activity predictor tool. A. Pathway activity prediction based on the microarray dataset. B. Pathway activity based on the NanoString dataset. Observed increases (red) and decreases (green) in mRNA abundance are indicated, as are predicted activation (orange) and inhibition (blue) of downstream targets. (TIFF) [file pone.0204197.s004.tiff]
